# A Comparison of Physician Pre-Adoption and Adoption Views on Electronic Health Records in Canadian Medical Practices

**DOI:** 10.2196/jmir.1726

**Published:** 2011-08-12

**Authors:** Norm Archer, Mihail Cocosila

**Affiliations:** ^1^DeGroote School of BusinessMcMaster UniversityHamilton, ONCanada; ^2^Athabasca UniversityAthabasca, ABCanada

**Keywords:** Electronic health record, information technology, medical practice, Canada

## Abstract

**Background:**

There is a major campaign involving large expenditures of public money to increase the adoption rate of electronic health record (EHR) systems in Canada. To maximize the chances of success in this effort, physician views on EHRs must be addressed, since user perceptions are key to successful implementation of technology innovations.

**Objective:**

We propose a theoretical model comprising behavioral factors either favoring or against EHR adoption and use in Canadian medical practices, from the physicians’ point of view. EHR perceptions of physicians already using EHR systems are compared with those not using one, through the lens of this model.

**Methods:**

We conducted an online cross-sectional survey in both English and French among medical practitioners across Canada. Data were collected both from physicians using EHRs and those not using EHRs, and analyzed with structural equation modeling (SEM) techniques.

**Results:**

We collected 119 responses from EHR users and 100 from nonusers, resulting in 2 valid samples of 102 and 83 participants, respectively. The theoretical adoption model explained 55.8% of the variance in behavioral intention to continue using EHRs for physicians already using them, and 66.8% of the variance in nonuser intention to adopt such systems. Perception of ease of use was found to be the strongest motivator for EHR users (total effect .525), while perceptions of usefulness and of ease of use were the key determinants for nonusers (total effect .538 and .519, respectively) to adopt the system. Users see perceived overall risk associated with EHR adoption as a major obstacle (total effect –.371), while nonusers perceive risk only as a weak indirect demotivator. Of the 13 paths of the SEM model, 5 showed significant differences between the 2 samples (at the .05 level): general doubts about using the system (*P* = .02), the necessity for the system to be relevant for their job (*P* < .001), and the necessity for the system to be useful (*P* = .049) are more important for EHR nonusers than for users, while perceptions of overall obstacles to adoption (*P* = .03) and system ease of use (*P* = .042) count more for EHR users than for nonusers.

**Conclusions:**

Relatively few differences in perceptions about EHR system adoption and use exist between physicians already using such systems and those not yet using the systems. To maximize the chances of success for new EHR implementations from a behavioral point of view, general doubts about the rationale for such systems must be mitigated through improving design, stressing how EHRs are relevant to physician jobs, and providing substantiating evidence that EHRs are easier to use and more effective than nonusers might expect.

## Introduction

### Context

Less than 25% of Canadian doctors use electronic health record (EHR) systems, less than in many other countries and ranking last among industrialized nations [1]. As a result, although almost all physicians use some form of computer support for scheduling and billing purposes, patient clinical records are mostly on paper, and are scattered and often inaccessible in doctors’ offices, clinics, test centers, labs, and hospitals.

An EHR (a term often used interchangeably with EMR, or electronic medical record) is a repository of information in computer-processable form that is employed by a physician to record and access information regarding the health of a patient. EHRs are becoming an essential artifact of current health care: to properly care for their patients, health care practitioners must have timely and accurate access to all relevant medical records. Absence of EHR systems can cause significant delays, duplication of effort, and even inaccuracy in diagnosing problems [2-4].

Despite the obvious advantages of EHR systems, their adoption rate has been slow in Canadian medical practices. Insufficient adoption of EHR systems is a highly complex problem that has not been addressed adequately in a comprehensive manner. Many interdependent factors influence adoption, and these must be considered simultaneously [5]. Above all, user perceptions are key in determining the success of an information technology (IT) deployment in any context [6].

Several studies regarding adoption of EHR systems in Canada and elsewhere have been done, but most of this work addressed in isolation a few relevant motivations for, or obstacles to, adoption [7-10]. It is important for such research to address the major issues comprehensively, or health care policies may be adopted that encourage the use of EHRs for the wrong reasons, leaving physicians with problems rather than benefits. The end result may be abandonment or underutilization of such systems. It is therefore important to have a broader understanding of physician views regarding salient factors for EHR adoption, and to compare how and why these views differ between adopters and nonadopters.

### Theoretical Background and Model

Investigating user adoption of new IT applications in various fields, including health care, is an established topic of information systems research. Various theories have been validated that attempt to better explicate user reasons to adopt new IT applications [11]. However, applying these models to medical practice situations is a challenging task because these practices involve a blend of autonomous physician activities with team interaction, thus requiring theories of both individual and organizational IT adoption. Therefore, one approach to examining medical practitioner perceptions of EHRs is to consider 2 sets of factors: (1) elements of individual use that stem from theories of individual decision making (eg, Technology Acceptance Model (TAM) [12] or Unified Theory of Acceptance and Use of Technology [11]), and (2) elements of organizational use (eg, the organizational version of TAM, TAM2 [13], or the Theory of Reasoned Action [14]).

Both of these categories of theories investigate primary factors such as behavioral intent to adopt and use an IT application, including performance expectancy (perceived usefulness) and effort expectancy (ease of use).

Of the many other (secondary) factors that may influence positively the intention to adopt EHRs, we consider 3 as more important: personal IT innovativeness (willingness of an individual to try new IT applications [15]), job relevance (functionalities provided for physician needs [16]), and social influence (influence of colleagues and significant others on adopting a system [17,18]).

A recent trend in information systems research is to also consider factors of resistance to a new IT implementation [19]. This category is of particular interest for investigating IT deployment in health care [20], as this field has high social sensitivity while lagging behind other industry sectors (eg, banking or tourism) in IT deployment. Factors having a negative effect on intention to adopt IT can be explained through the concept of perceived risk borrowed from consumer behavior, where consumers express doubts about purchasing due to anxieties such as wasting money and time, and privacy considerations [21]. Medical practitioners who consider adopting EHRs would tend to perceive these risks as being associated with themselves, even though the decision is in an organizational context, similarly to consumers considering the adoption of innovations [22].

Obstacles to EHR adoption are captured through the use of perceived overall risk, which expresses perceived negative consequences of EHR use. Recent information systems research has shown that perceived risk has several facets [21,23]. These depend on the context of the activity users would perceive as risky, but their aggregated influence should generate the same overall result. In the context of considering the adoption of EHRs, 3 risk facets are likely to be important antecedents of overall risk: (1) perceived performance risk (fear that the system will fail to perform as expected), (2) perceived legal and privacy risk (fear of legal and privacy problems from EHR use), and (3) perceived psychological risk (anxiety and stress about EHR implementation).

Building on previous consumer behavior and information systems research [19,24], we can find perceived overall risk to affect negatively both the perception of usefulness (hence performance expectancy) and the intention to use EHR systems. On the other hand, risk perception is alleviated when users perceive the system as easy to use [21].

**Figure 1:**
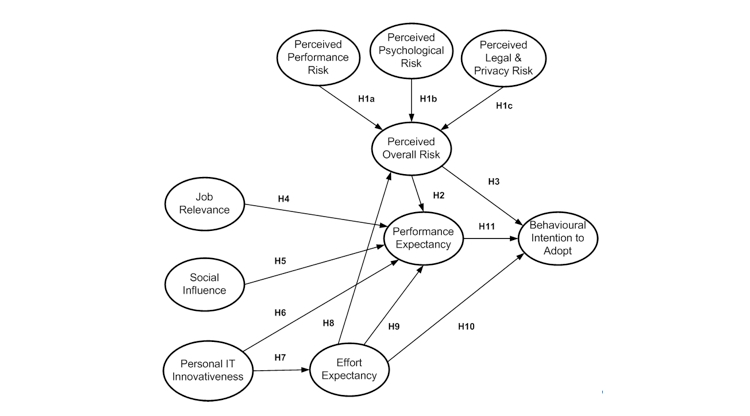
Theoretical model of electronic health record adoption in Canadian medical practices showing hypothesized (H) relationships

Based on the above theoretical reasoning and the results of previous research, the following theoretical model (Figure 1) and hypotheses are proposed:

*Hypothesis 1a) perceived performance risk will relate positively to perceived overall risk.*

*Hypothesis 1b) perceived psychological risk will relate positively to perceived overall risk.*

*Hypothesis 1c) perceived legal and privacy risk will relate positively to perceived overall risk.*

*Hypothesis 2) perceived overall risk will relate negatively to performance expectancy.*

*Hypothesis 3) perceived overall risk will relate negatively to behavioral intention to adopt.*

*Hypothesis 4) job relevance will relate positively to performance expectancy.*

*Hypothesis 5) social influence will relate positively to performance expectancy.*

*Hypothesis 6) personal IT innovativeness will relate positively to performance expectancy.*

*Hypothesis 7) personal IT innovativeness will relate positively to effort expectancy.*

*Hypothesis 8) effort expectancy will relate negatively to perceived overall risk.*

*Hypothesis 9) effort expectancy will relate positively to performance expectancy.*

*Hypothesis 10) effort expectancy will relate positively to behavioral intention to adopt.*

*Hypothesis 11) performance expectancy will relate positively to behavioral intention to adopt.*

### Purpose

This theoretical model captures the key behavioral factors that may favor or disfavor EHR adoption and use in Canadian medical practices, from the physicians’ point of view. Perceptions of physicians who have adopted EHRs can be systematically compared with those who have not, through the model’s lens.

## Methods

### Participant Recruitment and Data Collection

Data were collected through a cross-sectional online survey comprising an instrument based on the theoretical model hypotheses, together with relevant demographic questions. The items of all the constructs in the theoretical model were measured with 7-point Likert scales having as anchors *strongly disagree* and *strongly agree*. Relevant constructs were adapted from previous research in information systems, health care, and consumer behavior [11,14,20,21,23].

We designed 2 versions of the survey: one for physicians using EHR systems, and the other one for physicians not using such systems. The only difference between the 2 versions was that items capturing various perceptions for physicians using EHRs were formulated in the present tense (eg, “I find EHR useful”), while the questions addressed to the other category of physicians were phrased in the conditional present (eg, “I would find EHR useful”).

The survey was pretested by 4 PhD students in a Canadian university and, after improvement, pilot-tested with 3 practicing physicians. The research was approved by the Research Ethics Board of McMaster University.

Participants targeted for data collection were physicians working in medical practices in Canada. Because these health professionals have a busy schedule and are difficult to recruit for research, participant recruitment and survey administration were outsourced to a commercial firm having a panel of almost 67,000 preregistered physicians. The survey was conducted across Canada in both English and French, and participants were compensated for completing the survey. The total sample was of 220 participants with a balanced distribution between physicians working in clinics already using EHRs and physicians from practices not using EHRs. Additionally, an attempt was made to balance participants between those working in small (1 or 2 physicians) and larger clinics, and between general practitioners and specialists.

### Theoretical Model Analyses

Data were analyzed through descriptive statistics and structural equation modeling (SEM) techniques. A popular SEM approach, partial least squares (PLS), was used due to its suitability for predominantly exploratory research using complex models [25]. In addition, PLS requires no assumptions about sample data distributions [26] and works well with relatively small samples [27]. PLS analysis was done with SmartPLS software (release 2.0 [beta]; SmartPLS, Hamburg, Germany) in 2 stages: evaluation of the measurement model, followed by that of the structural model [28]. Demographic factors collected in the study were also tested as possible control variables in each of the 2 samples.

### Analysis of Differences between EHR Users and Nonusers

Outcomes of model analyses were compared through differences in path coefficients between the 2 models. The degree of difference was assessed with the *t* statistic with *N1* *+* *N2*– 2 degrees of freedom [29,30]:

*t* *=* *(* *Path1* *–* *Path* *2)* */* *[Spooled* * *sqrt* *(1* */* *N1* *+* *1* */* *N2)* *]*

where *Pa* *th1*, *Path* *2* are the corresponding path coefficients in the 2 models and *N1*, *N2* are the respective sample sizes.

*Spooled* is the pooled estimator for the variance, calculated as:

Spooled = sqrt {[ square of(N1 – 1)/(N1 + N2 – 2)] * square of SE1 +[square of (N2 – 1) / (N1 + N2 – 2)] *square of SE2}

where *S* *E* *1*, *S* *E* *2* are the standard errors of the respective model path coefficients.

The above approach is suitable when the variance of the 2 samples that are being compared is approximately the same, or when sample sizes are relatively large.

## Results

### Characteristics of Participants 

We collected 219 completed questionnaires: 119 EHR users and 100 nonusers. Eliminating questionnaires with more than 10% missing data left 102 and 83 valid responses, respectively. Missing data were replaced by the multiple imputation approach through a predictive mean matching procedure with 10 imputations [31]. The resulting corrected samples were the basis for subsequent statistical analyses.

Table 1 shows the demographic characteristics of the 2 samples used in the research.

**Table 1:** Participant characteristics

		EHR users	EHR nonusers
Sample size		102	83
Practice size			
	Small	47 (46%)	47 (57%)
	Large	55 (54%)	36 (43%)
Respondent practice type			
	General practice	50 (49%)	44 (53%)
	Specialists	52 (51%)	39 (47%)
Average medical experience (years)		18.5	20.7
Average number of physicians per practice		6.9	3.8
Work schedule			
	Full-time	99 (97%)	81 (98%)
	Part-time	3 (3%)	2 (2%)
Gender			
	Female	27 (26%)	15 (18%)
	Male	75 (73%)	68 (82%)

### Theoretical Model Analyses

A first evaluation of the measurement model indicated the necessity to drop 7 of 35 items from the EHR user model and 5 of 35 items from the EHR nonuser model because of unsatisfactory reliability and construct validity values. After rerunning SmartPLS for the remaining items, all constructs for both models displayed Cronbach alpha (composite reliability) values above .7, average variance extracted above .5, and item loadings above .7. Thus, the measurement model had acceptable reliability and convergent validity [32-34]. A visual inspection of the matrix of loadings and cross-loadings produced by SmartPLS indicated that the loadings of measurement items on their assigned constructs was larger than cross-loadings on other constructs, leading to the conclusion that constructs of both models had sufficient discriminant validity [28]. Overall, the statistical analysis of data for both models indicated adequate reliability and construct validity, leading to structural analysis of the model, the second step of PLS analysis.

**Figure 2:**
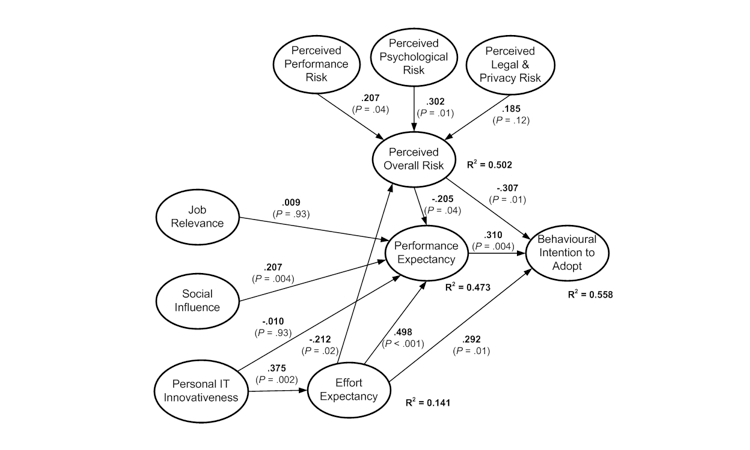
Structural evaluation of the electronic health record user model

**Figure 3:**
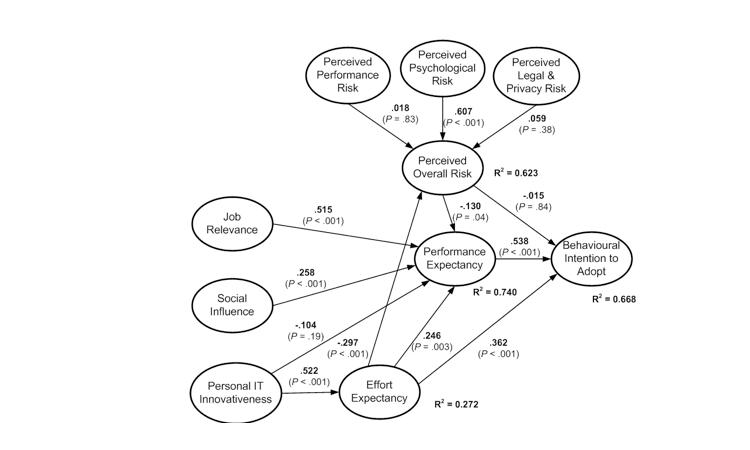
Structural evaluation of the electronic health record nonuser model

Evaluation of the structural model involved running SmartPLS with a bootstrap of 200 resamples. Results of path coefficients, their significance levels, and hypotheses outcomes, together with the values of the coefficient of determination, are shown in Figure 2 and Table 2 for the EHR users model and in Figure 3 and Table 3 for the EHR nonusers model.

**Table 2:** Hypothesis test results for the EHR user model

Hypothesis	Path	Coefficient	*P* value	Outcome
H1a	Performance risk → overall risk	.207	.04	Supported
H1b	Psychological risk → overall risk	.302	.01	Supported
H1c	Legal and privacy risk → overall risk	.185	.12	Rejected
H2	Overall risk → performance expectancy	–.205	.04	Supported
H3	Overall risk → behavioral intention	–.307	.01	Supported
H4	Job relevance → performance expectancy	.009	.93	Rejected
H5	Social influence → performance expectancy	.207	.004	Supported
H6	Personal IT^a^ innovativeness → performance expectancy	–.010	.93	Rejected
H7	Personal IT^a^ innovativeness → effort expectancy	.375	.002	Supported
H8	Effort expectancy → overall risk	–.212	.02	Supported
H9	Effort expectancy → performance expectancy	.498	<.001	Supported
H10	Effort expectancy → behavioral intention	.292	.01	Supported
H11	Performance expectancy → behavioral intention	.310	.004	Supported

^a^ Information technology.

**Table 3:** Hypothesis test results for the EHR nonuser model

Hypothesis	Path	Coefficient	*P* value	Outcome
H1a	Performance risk → overall risk	.018	.83	Rejected
H1b	Psychological risk → overall risk	.607	<.001	Supported
H1c	Legal and privacy risk → overall risk	.059	.38	Rejected
H2	Overall risk → performance expectancy	–.130	.04	Supported
H3	Overall risk → behavioral intention	–.015	.84	Rejected
H4	Job relevance → performance expectancy	.515	<.001	Supported
H5	Social influence → performance expectancy	.258	<.001	Supported
H6	Personal IT^a^ innovativeness → performance expectancy	–.104	.19	Rejected
H7	Personal IT^a^ innovativeness → effort expectancy	.522	<.001	Supported
H8	Effort expectancy → overall risk	–.297	<.001	Supported
H9	Effort expectancy → performance expectancy	.246	.003	Supported
H10	Effort expectancy→ behavioral intention	.362	<.001	Supported
H11	Performance expectancy → behavioral intention	.538	<.001	Supported

^a^ Information technology.

SmartPLS results also provided the total effects of the factors in the theoretical model on behavioral intention to use EHRs, for the 2 categories of participants (see Table 4).

Number of physicians per medical practice, years of medical experience, and gender of the respondents were tested as potential control variables. Path coefficients and significance levels for the paths from these constructs to the endogenous latent variables of both theoretical models were calculated by running PLS analysis. A fourth potential control variable (work schedule) did not produce any results in the PLS analysis due to its heavy bias: over 97% of the physicians in both samples reported working full time. Of all the control variables tested, only the number of physicians per practice in the EHR nonusers model was found to be significant, with a path coefficient of .106 (*P* = .04) to effort expectancy. The variance explained by this variable increased from *R* ^2^ = 0.272 to *R* ^2^ = 0.283.

**Table 4:** Total effects and their significance levels on behavioral intention

Antecedent construct	EHR users	EHR nonusers
Perceived performance risk	–.077 (*P* = .048)	–.002 (*P* = .88)
Perceived psychological risk	–.112 (*P* = .07)	–.052 (*P* = .28)
Perceived legal and privacy risk	–.068 (*P* = .17)	–.005 (*P* = .61)
Perceived overall risk	–.371 (*P* < .001)	–.085 (*P* = .27)
Job relevance	.003 (*P* = .94)	.277 (*P* < .001)
Social influence	.064 (*P* = .06)	.139 (*P* < .001)
Personal IT^a^ innovativeness	.194 (*P* = .03)	.215 (*P* = .005)
Effort expectancy	.525 (*P* < .001)	.519 (*P* < .001)
Performance expectancy	.310 (*P* = .004)	.538 (*P* < .001)

^a^ Information technology.

### Analysis of Differences Between EHR Users and Nonusers

The standard deviations of the corresponding path coefficients of the 2 models are of similar orders of magnitude in most cases. In addition, sample sizes were relatively large, at more than twice the minimum sample required for PLS [34]. Accordingly, the *t* statistic formula described above was used to calculate the degree of difference between the path coefficients of the 2 models. The results are presented in Table 5.

**Table 5:** Statistical analysis of differences between EHR users and nonusers

Path	EHR users path coefficient	EHR nonusers path coefficient	*t* Value of difference	*P* value
Performance risk → overall risk	.207	.018	1.444	.07
Psychological risk → overall risk	.302	.607	2.034	.02
Legal and privacy risk → overall risk	.185	.059	0.874	.19
Overall risk → performance expectancy	–.205	–.130	0.618	.27
Overall risk → behavioral intention	–.307	–.015	1.964	.03
Job relevance →> performance expectancy	.009	.515	3.600	<.001
Social influence → performance expectancy	.207	.258	0.544	.29
Personal IT^a^ innovativeness → performance expectancy	–.010	–.104	0.685	.25
Personal IT^a^ innovativeness → effort expectancy	.375	.522	0.982	.16
Effort expectancy → overall risk	–.212	–.297	0.713	.24
Effort expectancy → performance expectancy	.498	.246	1.733	.042
Effort expectancy → behavioral intention	.292	.362	0.495	.31
Performance expectancy → behavioral intention	.310	.538	1.659	.049

^a^ Information technology.

## Discussion

### Principal Findings and Comparison with Prior Work

The objectives of this study were to identify the main factors influencing the adoption of EHRs in Canada from the medical practitioners’ perspective, and to compare the views of physicians already using such systems with those not using them. The most important findings in general are that high performance expectancy and little effort expectancy about EHRs are significant positive adoption factors, while perceived overall risk is a deterrent. Few differences in the perceptions of EHR systems were noticed between users and nonusers: these concern mostly the adoption roles of effort expectancy and perceived overall risk. The following focuses first on the results from existing users, then on results from nonusers, and then on a comparison of differences between the 2 population samples.

#### EHR User Model

As expected, according to a large body of information systems literature, usefulness perception captured through performance expectancy is a key direct reason influencing physicians already using EHRs to continue using them [6] (Figure 2). Of the hypothesized antecedents of performance expectancy, only social influence proved to have a significant influence. Thus, in a sensitive field like health care, positive references from colleagues and collaborators about EHRs is a factor influencing practitioners to see the usefulness of such systems. This finding is consistent with previous organizational information systems studies that include subjective norm in technology adoption models [13].

Effort expectancy, indicating a perception of ease of use, is an important determinant of both behavioral intention to adopt and performance expectancy, in agreement with information system studies [6]. Moreover, effort expectancy has by far the strongest total effect on behavioral intention (coefficient = .525, *P* < .001), due to its direct path as well as its indirect influence through performance expectancy (Figure 2 and Table 4): ease of use amplifies the perception of usefulness and, hence, intention to continue using the system. Ease of use is augmented by physician personal IT innovativeness, which is concordant with the literature [15,35]: physicians interested in IT innovations have little difficulty in using EHRs, thus reinforcing the perception of usefulness and intention to use. Personal IT innovativeness has a significant overall effect (*P* = .03) on behavioral intention to use EHRs (Table 4).

With a negative effect of –.371 (*P* < .001), perceived overall risk is the main obstacle to intention to continue using EHRs, for physicians already using them (Figure 2 and Table 4). This is consistent with consumer behavior research where perceived risk associated with a product negatively influences intention to purchase it [24]. Of the 3 types of risk considered meaningful in this study, only perceived performance risk (*P* = 0.4) and, especially, perceived psychological risk (*P* = .01) had a significant influence on the overall risk perceived by EHR users. This shows the key deterrent roles of fears about the system not working as expected and general doubts about the role played by EHRs in medical practices. These findings are concordant with previous consumer behavior and information systems studies [19,21]. As shown in Figure 2, a low expectancy of effort eases the perception of risk (coefficient = –.212, *P* = .02). This demonstrates the twofold role played by effort expectancy in the model (a positive direct and indirect influence on one side and a risk-reducing effect on the other side). Consequently, one way to promote more effective use of EHR systems among physicians already using them is to focus on improving ease of use.

Overall, the theoretical model explaining the adoption of EHR by physicians using these systems had a moderately high *R* ^2^ (variance explained) values for all the endogenous constructs (between 0.141 for effort expectancy and 0.558 for behavioral intention) and a high proportion of significant paths: 10 out of 13 (Table 2). According to the literature on PLS methodology, the proposed model could be termed appropriate [36]. 

#### EHR Nonuser Adoption Model

Similarly to the model for physicians using EHRs, performance expectancy and effort expectancy are strong and significant explanations of behavioral intention to adopt for nonusers (Figure 3). Results in Table 4 show their total effect is about the same level (.538, *P* < .001 and .519, *P* < .001, respectively), demonstrating that it is important for EHRs to be perceived as both useful and easy to use by physicians who have not yet been exposed to this technology. While influences from the social environment are a significant element strengthening perceptions of usefulness, physician IT innovativeness remains a strong antecedent of perceptions of ease of use (Table 3). In addition, nonusers feel that it is important for EHRs to be relevant in their jobs when they decide on adoption (Figure 3 and Table 4).

Perceived overall risk remains a negative factor in the adoption equation for EHR nonusers, but with a reduced influence compared with results from the user model. There is a direct negative effect on the usefulness perception (*P* = .04) but no significant direct or total effect on the intention to adopt such systems (Figure 3 and Table 4). Perceived psychological risk is the only antecedent of overall risk that is significant in the model, but its influence is strong (coefficient = .607, P < .001) (Table 3). A possible explanation is that nonusers do not have a deep understanding of the potential obstacles associated with the use of such systems. Nonetheless, anxiety about introducing an EHR system is an important obstacle that needs to be mitigated if adoption is to be encouraged.

Of the 4 endogenous constructs of the nonuser model, 3 displayed moderately high *R* ^2^ values (between 0.272 and 0.740) and 9 of the 13 paths hypothesized were significant. Hence, the model explains relatively well the nonuser perceptions of EHR adoption. Therefore, the theoretical model appears to be appropriate for nonusers [36], for reasons similar to the suitability demonstrated for the user model.

#### Control Variable Tests

The control variables tested (number of physicians per medical practice, years of medical experience, work schedule, and gender of the respondents) did not produce any effect, with one exception: the positive influence of the number of physicians on effort expectancy for EHR nonusers. The moderately strong and significant effect may indicate that the larger the clinic, the lower the effort perceived in adopting and using EHRs. This effect is likely due to the availability of more technologically knowledgeable support staff in larger clinics.

#### Differences in Perceptions Between EHR Users and Nonusers

The models depicted in Figures 2 show differences in factor influences between EHR users and nonusers. However, results in Table 5 indicate significant differences in only 5 paths out of 13. Thus, perceived psychological risk is a strong and significant antecedent of perceived overall risk in both models, but its influence is much higher for nonusers (*P* = .02). Therefore, decreasing perceived adoption obstacles and easing the acceptance of EHRs among physicians not yet using them would help to address general doubts about the rationale of using EHRs. However, perceived overall risk is a significantly stronger obstacle for users than for nonusers (*P* = .03), indicating that user concerns must be addressed during implementation to avoid negative views in later stages.

As shown in Table 5, job relevance is a much stronger antecedent of performance expectancy for EHR nonusers than for users (*P* < .001). This indicates that, in the process of developing and implementing EHRs for practices not yet using them, it is important that these applications address the most important tasks as seen by physicians.

Effort expectancy has a more significant influence on performance expectancy for EHR users than for nonusers (*P* = .042, in Table 5). This indicates that the ease of use perceived by physicians currently using EHRs is higher than for nonusers. Therefore, the design and implementation process of new EHRs for medical practices should include efforts to mitigate concerns about the difficulty of using the systems. 

Performance expectancy is an important antecedent of intention to use in both models. Its influence appears to be stronger for nonusers but only at the limit of significance (*P* = .049).

### Limitations

This study has certain limitations. Although study participants were recruited from a large sampling frame, they self-selected. Further, because of feasibility constraints, the resulting sample size was relatively small for such a complex investigation. Although the valid samples of 102 EHR users and 83 EHR nonusers were more than twice as large as the minimum PLS requirements for a reliable statistical analysis [34,37], samples were not particularly homogeneous from a medical practice point of view. This was demonstrated in part by comments received from participants that, although difficult to classify, ranged all the way from frustration to satisfaction for users, and from concerns about productivity to positive anticipation from nonusers.

Although the study showed little effect from participant characteristics (years of medical experience, gender, full time vs part time), there was some differentiation based on clinic size (number of physicians employed), and future research should use larger samples that can discriminate more between such characteristics. Larger samples might also differentiate between physicians involved versus those not involved in decisions on EHR system adoption. For example, various risk perceptions might depend on the degree of respondent involvement in EHR adoption decisions.

This study did not attempt to differentiate between various levels of EHR experience, frequency of use, and complexity of tasks performed with the system by EHR users. Thus, physicians in some clinics might use only basic functionalities of EHRs (eg, tracking patient prescription records) that would perform well but have less impact on work quality and productivity, while physicians in other practices may use more complex functions, better suited to supporting their tasks, but encountering different types of user problems.

For nonusers, a useful future differentiation would be between the medical practices that have not seriously considered adopting EHRs yet and those practices that are seriously considering the use of EHRs within a certain time horizon. Perceptions of certain risks may differ between these 2 categories, depending on physician familiarity with related issues.

### Conclusions and Practical Implications

Comparing EHR perceptions of physicians already using the systems with those not yet using them through a rigorous theoretical approach has helped to understand certain key behavioral aspects that must be addressed in the deployment of these systems. To maximize the chances of success of new EHR implementations, it is necessary to focus on mitigating doubts about the rationale for such systems, to stress how EHRs are relevant for physician jobs, and to demonstrate that they are easier to use than nonusers might expect. This cannot be done without involving the end users in the design and evolution of EHRs, so they present highly usable and easy-to-learn interfaces and support decision-support capabilities that are needed by physicians. Further research is expected to deepen the findings from this study. Understanding physician perceptions of EHRs is a critical issue that must be addressed before better systems can be designed and adopted by the medical community. 

## Abbreviations

EHR: electronic health record
IT: information technology
PLS: partial least squares
SEM: structural equation modeling
TAM: Technology Acceptance Model
